# Patient Portals as Facilitators of Engagement in Patients With Diabetes and Chronic Heart Disease: Scoping Review of Usage and Usability

**DOI:** 10.2196/38447

**Published:** 2023-08-25

**Authors:** Benjamin Scheckel, Katharina Schmidt, Stephanie Stock, Marcus Redaèlli

**Affiliations:** 1 Institute of Health Economics and Clinical Epidemiology Faculty of Medicine and University Hospital Cologne University of Cologne Cologne Germany

**Keywords:** patient portal, eHealth, online platform, usability, feasibility, diabetes, heart disease

## Abstract

**Background:**

Patient portals have the potential to improve care for chronically ill patients by engaging them in their treatment. These platforms can work, for example, as a standalone self-management intervention or a tethered link to treatment providers in routine care. Many different types of portals are available for different patient groups, providing various features.

**Objective:**

This scoping review aims to summarize the current literature on patient portals for patients with diabetes mellitus and chronic heart disease regarding usage behavior and usability.

**Methods:**

We conducted this review according to the PRISMA (Preferred Reporting Items for Systematic Reviews and Meta-Analyses) statement for scoping reviews. We performed database searches using PubMed, PsycInfo, and CINAHL, as well as additional searches in reviews and reference lists. We restricted our search to 2010. Qualitative and quantitative studies, and studies using both approaches that analyzed usage behavior or usability of patient portals were eligible. We mapped portal features according to broad thematic categories and summarized the results of the included studies separately according to outcome and research design.

**Results:**

After screening, we finally included 85 studies. Most studies were about patients with diabetes, included patients younger than 65 years, and were conducted in the United States. Portal features were categorized into educational/general information, reminder, monitoring, interactivity, personal health information, electronic/personal health record, and communication. Portals mostly provided educational, monitoring, and communication-related features. Studies reported on usage behavior including associated variables, usability dimensions, and suggestions for improvement. Various ways of reporting usage frequency were identified. A noticeable decline in portal usage over time was reported frequently. Age was most frequently studied in association with portal use, followed by gender, education, and eHealth literacy. Younger age and higher education were often associated with higher portal use. In two-thirds of studies reporting on portal usability, the portals were rated as user friendly and comprehensible, although measurement and reporting were heterogeneous. Portals were considered helpful for self-management through positive influences on motivation, health awareness, and behavioral changes. Helpful features for self-management were educational/general information and monitoring. Barriers to portal use were general (eg, aspects of design or general usability), related to specific situations during portal use (eg, login procedure), or not portal specific (eg, user skills and preferences). Frequent themes were aspects of design, usability, and technology. Suggestions for improvement were mainly related to technical issues and need for support.

**Conclusions:**

The current state of research emphasizes the importance of involving patients in the development and evaluation of patient portals. The consideration of various research designs in a scoping review is helpful for a deeper understanding of usage behavior and usability. Future research should focus on the role of disease burden, and usage behavior and usability among older patients.

## Introduction

Type 2 diabetes mellitus (DM) and chronic heart disease, especially coronary heart disease (CHD), remain major health issues in the 21st century. According to World Health Organization estimations, 1.6 million deaths worldwide were directly caused by diabetes [1]. Furthermore, according to the global burden of disease study, about 9 million deaths worldwide were attributed to CHD, making it the leading cause of death [2]. These conditions require ongoing self-management to minimize complications and progression. Patients with diabetes, for example, need to monitor blood glucose levels, take medications often, and generally change their lifestyle [3]. They continuously need to consider these aspects in their daily lives and therefore need to be well organized. Thus, self-management skills are an important condition to improve care. However, chronically ill patients often fail to reach adequate self-management for various reasons [4]. There are several interventions to help patients manage their disease, including patient education and collaborative care models [4]. Interventions to improve self-management in patients with diabetes and chronic heart disease have the potential to reduce the burden of the disease and health care costs [5-9].

eHealth applications have increasingly come into focus when talking about self-management of chronic diseases. In addition, the COVID-19 pandemic has led to a significant global acceleration of digitalization in the health sector and thus to an increase in the importance of eHealth applications in routine care. For example, preliminary data from Germany for the year 2020 show a strong increase in the use of telemedicine services in addition to a significant decrease in doctor visits [10]. This highlights the increasing importance of supplementary interfaces between health care professionals and patients.

Patient portals, as a gateway to online health applications, can enable patients to better participate in their care and thus better self-manage it. Often, patient portals act as interfaces to electronic health records (EHRs) or personal health records (PHRs). Besides, patient portals without fixed tethering to patient data have been developed for patient use [11]. In our review, we define a patient portal as a web-based interface that provides access to various health-related features for specific or all patients within the scope of care. The types of features can vary, but all have the focus of allowing patients to participate in their care and thus promote their disease self-management. Possible features include secure messaging with care providers or peers, provision of educational or personal health information, and monitoring [12]. The functional scope depends on the focus of the portal, for example, a patient portal tethered to an EHR or a specially developed portal as a standalone self-management intervention. In the context of this review, the term “patient portal” is used throughout. Since the terms “eHealth” and “digital health” are the most used umbrella terms for different digital applications in the context of health care and their user interfaces, we did not rely on them.

Several reviews have already comprehensively addressed the usability and clinical effectiveness of patient portals linked to EHRs [12-14]. An umbrella review by Antonio et al [13] showed that patient portals linked to EHRs generally tend to be used more by younger men and those with higher education or health literacy. Portal use (for portals tethered to EHRs) is also reported to be greater among patients with higher disease burden [14]. These portals also focus on retrieving personal health information, while telerehabilitation portals primarily focus on monitoring or tracking [15]. In terms of usability, the simplicity of the portal, the presence of a communication function, a simple presentation, and the use of simple language are important from the user perspective [13]. Talking about older users, support in using eHealth applications is often cited as a facilitator, whereas lack of time, monetary costs, and lack of motivation appear to be barriers in using those tools [16]. Regarding various chronic diseases, patients with diabetes are often addressed in existing reviews [17,18]. For this user group, it has been shown that a lack of user friendliness of portals and security concerns are important barriers [17]. Improvements were seen in portal users with respect to certain laboratory values, such as HbA_1c_, but not for other clinical outcomes [13]. In addition, patient portals that allow access to EHRs have the potential to improve patient engagement, satisfaction, and patient-provider communication [13,14].

However, existing reviews mostly focus on a single type of patient portal, especially those tethered to EHRs, and do not cover the existing range of patient portals. Additionally, summarizing studies from different countries or rather cultures with different attitudes and opportunities in the area of health technologies is, in our opinion, better handled by a scoping review approach. Therefore, we decided to take a step back and use this approach to capture the breadth of research on patient portals for patients with diabetes or chronic heart disease. In addition, beyond the clinical effectiveness of patient portals, the need to further investigate aspects of use and usability continues to emerge [19]. The specific objectives of our scoping review were therefore to (1) identify what types of patient portals currently exist for patients with diabetes and chronic heart disease, and which features they provide; (2) describe the usage behavior of patients and the associated factors; and (3) map how patients evaluate usability in the context of their self-management.

## Methods

### Review Type

We chose a scoping review approach for 2 reasons. First, we expected different types of patient portals, which may only be comparable to a limited extent (depending on the area of application and health care system). Second, since patient portals are an increasingly recognized topic in health science, which already resulted in many different scientific approaches, scoping reviews allow to include and consider such a large body of literature and the elaboration of core aspects [20].

We conducted this review in accordance with the PRISMA (Preferred Reporting Items for Systematic Reviews and Meta-Analyses) statement for scoping reviews [21]. The PRISMA-ScR (PRISMA extension for Scoping Reviews) checklist can be found in Multimedia Appendix 1. We have not registered this review or published a protocol for it.

### Eligibility Criteria

We developed broad inclusion criteria for this review, in line with the basic idea of a scoping review to generate a comprehensive overview. Qualitative or quantitative studies, or studies using both approaches integratively or separately (mainly known as mixed-methods studies) were eligible for inclusion. The population included patients with type 2 DM or CHD who were older than 18 years of age. If studies also included subjects with type 1 DM, and thus possibly younger patients, the studies had to have at least a 50% proportion of patients with type 2 DM. Since our preliminary searches showed various terms for CHD, we used search terms for general chronic heart disease or cardiovascular disease. We also included patients with “heart failure” because it is likely that a substantial proportion of CHD patients already have heart failure at the diagnosis of CHD [22]. Among studies that included patients with different diseases, we excluded studies with less than 50% of patients with DM or cardiac disease.

Studies were included if they evaluated a patient portal to improve disease self-management from the patient perspective. Portals could be disease-specific or rather generic as long as patients with DM or cardiac disease were sufficiently represented. Descriptions of portal development only, without at least reported piloting, were excluded. PHRs were taken into account, provided that data on use or usability were reported. Furthermore, we excluded studies examining portals provided exclusively as mobile health applications.

The outcomes of interest were usage behavior and usability, including barriers and facilitators, and the associations with portal use. We captured “usage behavior” as an operationalization of the feasibility and acceptability of online portals. User satisfaction as an aspect of usability was also considered. Because our outcomes of interest represented “soft” outcomes that may appear in studies with different labels, we grouped studies thematically according to their outcomes as part of the data charting process. If a study reported the clinical outcomes of a portal in addition to the outcomes we intended, we only extracted the outcomes of interest from these studies.

### Search Strategy

We performed systematic database searches using PubMed, PsycINFO, and CINAHL. The search strategy consisted of a combination of terms for (1) “patient portal,” (2) “diabetes” or “chronic heart disease” including “coronary heart disease,” and (3) usage, usability, feasibility, and acceptability. We additionally performed extensive hand searches using different additional search platforms (eg, Google Scholar and IEEE Xplore), databases, and journals. Furthermore, clinical trial registers (ClinicalTrials.gov and DRKS) were searched. The references of included studies were searched to identify further studies and cross-validate the results of the search. BS and KS developed the search strategy. MR checked and revised the strategy. The full search strategies for each database as well as details on further searches are provided in Multimedia Appendix 2. We only included full-text articles from peer-reviewed journals, published in German or English. We restricted our search to January 2010. The last search was carried out on November 30, 2022.

### Selection of the Literature

We collected and screened identified records using the reference management tool EndNote. We identified and excluded duplicates. BS and KS screened and selected the remaining records independently of each other. Disagreements were resolved in discussion with MR. We recorded details of the selection process and filled them in a PRISMA literature flowchart according to Moher et al [23]. In addition, we reported reasons for full-text exclusion.

### Data Charting

We developed a data extraction sheet, and checked and revised it. Among other items, the data extraction form included information on general study characteristics, participants, evaluated portals, and quantitative and qualitative results.

KS and BS performed data extraction, and MR checked it. We did not perform a quality assessment of the included studies, since this is generally not performed in a scoping review [20]. We have addressed research design–specific strengths and limitations in the discussion.

### Synthesis of the Included Studies

In accordance with the methodology of a scoping review and the expected variety of included studies, we did not conduct a meta-analysis or formal meta-synthesis. We divided our synthesis into 2 major sections. First, we described the general characteristics of the included studies narratively. This included descriptions of the identified portals. For the synthesis of portal features, we used the extracted information on the portals to categorize the identified features. Second, quantitative and qualitative studies, and studies following both approaches were grouped according to the reported outcomes and were summarized narratively mainly following a convergent segregated approach [24]. In addition to the aspects of usage behavior and usability, we also summarized whether portal use was considered helpful for self-management from the patient’s perspective. In order to prepare the narrative synthesis of the qualitative studies, we examined these broadly by superordinate themes in relation to the outcomes of interest. In line with a convergent segregated mixed-methods approach, the results of the qualitative studies were compared with those of the quantitative studies for each outcome to identify gaps in both research designs.

## Results

### Search Results

**Figure 1:**
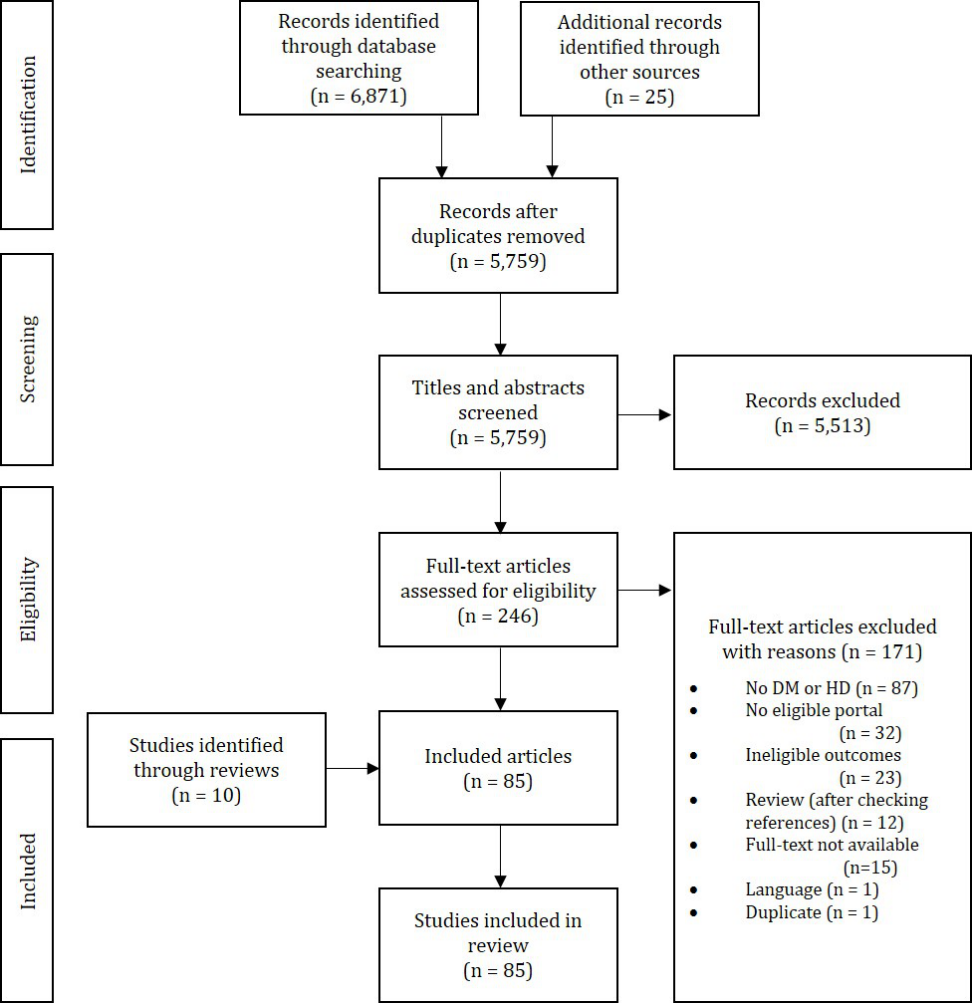
Study selection process. DM: diabetes mellitus; HD: heart disease.

In total, we identified 6871 publications via database searches and 25 via supplementary hand searches. After removal of duplicates, 5759 publications remained for further screening. Of these, we excluded 5513 records based on title and abstract screening. Thus, for 246 potentially relevant articles, we obtained and screened the full texts. The screening of these led to the exclusion of 171 full-text articles. The reasons for exclusion were mainly the population, the lack of a portal, and inappropriate outcomes (Figure 1). Screening of identified reviews led to the inclusion of 10 additional articles. Thus, we finally included 85 studies in our scoping review (Figure 1).

### Characteristics of the Included Studies

Of the 85 included studies, 38 were quantitative studies, 12 were qualitative studies, and 35 were mixed-methods studies. Among the quantitative studies, 13 were randomized controlled trials.

Most of the included studies were from the United States (n=42). There were 11 studies from the Netherlands; 6 from Canada; 5 each from the United Kingdom and Scotland; 4 from Australia; 2 each from Denmark and Germany; and 1 each from Russia, Italy, Taiwan, Korea, China, France, Belgium, and New Zealand.

In total, the 85 studies included 7 to 55,605 participants, with 1 study not reporting a number. Thirteen studies did not report the mean age of the participants. The mean age in the remaining studies ranged from 42.4 to 70.8 years. In 11 studies, the average age of the participants was over 65 years.

Participants with DM were present in 57 of the 85 studies. Participants with chronic heart disease were present in 16 studies, and participants with unspecified cardiovascular disease were present in 5 studies. In 7 studies, participants with both DM and heart or cardiovascular disease were included.

Thirteen studies recorded whether access to a computer (or tablet or similar) was available and its use, and an average of 85.1% (689/810) of study participants had access. In 2 of these 13 studies, computer use averaged about 11 hours per week. Moreover, 15 studies described the participants’ internet access and use. Approximately 88.0% (1885/2142) of the study participants reported access to the internet. In 5 of these 15 studies, 70.0% (771/1101) of the participants stated that they used the internet at least once a week. However, some respondents also indicated daily use.

Twelve studies examined computer literacy; however, various measurement instruments were used. In summary, 8 studies indicated the computer literacy of the participants as good or described the users as “experienced.” Three studies reported the status of the vast majority of participants as beginners, and 1 study had no clear distribution.

Of the 11 studies with an average participant age over 65 years, 2 provided information on the assessment of technical affinity, and the majority of participants had internet access at home and were experienced users.

### Portal Characteristics

The terms for the portals and their appearances varied widely. The most common term used was “portal,” including patient portal, web portal, internet portal, and online portal. The terms “program” and “platform” were also used frequently. There were also conceptual links of portals and EHRs/PHRs, for example, when a patient portal was tethered to an EHR or PHR. Other terms used were virtual environment, web application, and PHR.

A total of 38 studies reported on a portal that was specifically targeted at patients with DM. In 15 studies, portals were designed exclusively for patients with heart disease. Portals with no specific disease orientation or target group were present in 23 studies. The remaining 9 portals targeted either specific populations, such as low-income individuals, or subgroups of the included diseases.

The thematic analysis revealed 7 summary categories of portal features that were not completely mutually exclusive. We named these categories as follows: educational information, reminder, monitoring, interactivity, personal health information, EHR/PHR, and communication. Detailed descriptions of the functions can be found in Table 1.

Table 2 provides an overview of the included studies, and the respective portals and their features as reported [25-109]. One study did not describe the portal features. Two studies evaluated user requirements for a portal and portal features that were not yet existing, and therefore, they did not provide information on portal features (Table 2). Providing educational and general information was the most common feature (63 studies), followed by communication (45 studies) and monitoring (40 studies).

**Table 1:** Descriptions of identified portal features.

Portal feature	Description	Examples
Educational/general information	Provision of knowledge Disease-specific or general information	Patient training courses Link list to nutritional information
Reminder	Notifications	Goal setting Appointment reminders
Monitoring	Input of own parameters with progress monitoring Risk assessment for complications or cardiovascular events	Entering blood values such HbA_1c_ or blood glucose
Interactivity	Possibility to design elements interactively	Customizable infographics
Personal health information	Retrieval of individual pieces of personal health information	Medical history Medication list Laboratory values Treatment plan
EHR^a^/PHR^b^	Portal acts as an interface to a health record	Patient portal with access to medical records Portal including only data entered and held by a patient (PHR) Hybrid portal with access to medical records and supplementation with own data (eg, MDMW^c^ online portal)
Communication	Any form of enabled communication Messages via the portal’s own application or secure data exchange	Possibility to make appointments Discussions with peers via a forum

^a^EHR: electronic health record.

^b^PHR: personal health record.

^c^MDMW: My Diabetes My Way.

**Table 2:** Characteristics of the included studies.

Study ID	Research design	Country	Sample size	Age (years), mean	Disease	Portal features						
						Edu^a^	R^b^	M^c^	Int^d^	PHI^e^	EHR/PHR^f^	C^g^
Brennan, 2010 [25]	Quantitative	USA	282	64	HD^h^	+^i^	−^j^	+	+	−	−	+
Cho, 2010 [26]	Quantitative	USA	201	58.9	DM^k^	+	+	+	−	−	−	+
Kerr, 2010 [27]	Mixed methods	UK	168	66.8-71^l^	HD	+	−	−	+	−	−	−
Noh, 2010 [28]	Quantitative	ROK^m^	40	42.4	DM	+	−	−	−	−	−	−
Sarkar, 2010 [29]	Quantitative	USA	14,102	59	DM	+	−	−	−	+	−	+
Glasgow, 2011 [30]	Quantitative	USA	270	60	DM	+	+	+	+	+	−	+
Mayberry, 2011 [31]	Mixed methods	USA	45	58	DM	−	−	−	−	+	+	+
Nijland, 2011 [32]	Mixed methods	Netherlands	50	61	DM	+	+	+	−	−	−	+
Segall, 2011 [33]	Mixed methods	USA	23	53	CVD^n^	−	−	−	−	+	+	−
Shaw, 2011 [34]	Quantitative	USA	5963	52	DM	−	+	−	−	+	−	−
Chau, 2012 [35]	Mixed methods	China	100	61.5	DM	+	−	+	−	−	−	−
Heinrich, 2012 [36]	Mixed methods	Netherlands	564	57	DM	+	+	−	−	−	−	−
Jethwani, 2012 [37]	Mixed methods	USA	75	58-61^l^	DM	+	−	+	−	−	−	+
Urowitz, 2012 [38]	Qualitative	Canada	854	NR^o^	DM	+	−	+	+	−	+	−
Clark, 2013 [39]	Mixed methods	Australia	27	62.4	HD	+	−	+	−	−	−	+
Osborn, 2013 [40]	Mixed methods	USA	75	56.9	DM	+	−	−	−	+	+	+
Ryan, 2013 [41]	Quantitative	USA	21	54.36	DM	+	−	−	−	−	−	+
Wade-Vuturo, 2013 [42]	Mixed methods	USA	54	57.1	DM	−	−	−	−	−	+	+
Bartlett, 2014 [43]	Mixed methods	UK	7	NR	HD (HF^p^)	+	−	+	−	−	−	−
Johnson, 2014 [44]	Mixed methods	USA	20	53	DM	+	−	−	+	−	−	−
Lau, 2014 [45]	Quantitative	Canada	157	54.7	DM	+	−	−	−	+	−	+
Roelofson, 2014 [46]	Quantitative	Netherlands	1378	64.1	DM	+	+	+	−	+	−	−
Ronda, 2014 [47]	Quantitative	Netherlands	1390	63.9	DM	+	−	+	−	+	+	+
Rosal, 2014 [48]	Quantitative	USA	89	52	DM	+	−	−	+	−	−	−
Ruggiero, 2014 [49]	Quantitative	USA	41	55.2	DM	+	−	−	−	−	−	−
Sieverink, 2014 [50]	Quantitative	Netherlands	161	NR	DM	+	+	+	−	+	−	−
Yu, 2014 [51]	Qualitative	Canada	23	NR	DM	+	+	+	−	−	−	−
Clark, 2015 [52]	Mixed methods	Australia	5	61.6	HF	+	−	+	−	−	−	−
Eschler, 2015 [53]	Qualitative	USA	34	56	DM	−	−	−	−	+	−	+
Fuji, 2015 [54]	Qualitative	USA	59	59	DM	+	−	+	−	+	+	+
Jones, 2015 [55]	Quantitative	USA	2282	NR	HD, CVD, DM	−	+	+	−	+	+	+
Payne, 2015 [56]	Mixed methods	Canada	7	57	CVD (HF)	+	+	−	−	−	−	−
Ronda, 2015 [57]	Quantitative	Netherlands	632	59.7	DM	+	−	+	−	+	+	+
Tieu, 2015 [58]	Qualitative	USA	11	57	DM, CVD	+	−	+	−	−	−	−
Wakefield, 2015 [59]	Mixed methods	USA	9	70	HD (HF)	−	−	+	−	−	−	−
Hofmann, 2016 [60]	Mixed methods	UK	19	63.5	DM	+	−	−	+	−	+	+
Kopanitsa, 2016 [61]	Qualitative	Russia	36	63.7	DM	−	−	+	+	−	+	−
Nelson, 2016 [62]	Mixed methods	USA	32	51.7	DM	+	−	−	−	−	−	+
Toscos, 2016 [63]	Quantitative	USA	200	NR	HD	+	+	+	−	+	+	+
Van Vugt, 2016 [64]	Quantitative	Netherlands	132	67.9	DM	+	+	+	−	+	−	−
Wake, 2016 [65]	Quantitative	UK	NR	NR	DM	+	+	+	−	+	+	+
Connelly, 2017 [66]	Mixed methods	UK	61	63-66.7^l^	DM	+	+	−	+	−	−	−
Hansel, 2017 [67]	Quantitative	France	120	56.5	DM	+	+	+	−	−	−	−
Hart, 2017 [68]	Quantitative	Netherlands	282	63	DM	−	+	+	−	−	−	−
Higgins, 2017 [69]	Mixed methods	Australia	21	62	CHD^q^	+	−	+	−	−	−	−
Huang, 2017 [70]	Quantitative	USA	100	74	DM	+	−	+	+	−	−	−
Johnson, 2017 [71]	Mixed methods	USA	10	58	DM	+	−	−	+	−	−	+
Shaw, 2017 [72]	Mixed methods	USA	14	60	CVD	+	−	−	−	−	−	−
Sieck, 2017 [73]	Qualitative	USA	29	NR	CVD	−	−	−	+	+	−	+
Srinivas, 2017 [74]	Mixed methods	USA	5	61.2	CHD (HF)	+	+	+	−	−	−	−
Tieu, 2017 [75]	Mixed methods	USA	25	58	DM, HD, CVD	+	−	−	−	−	−	−
Wakefield, 2017 [76]	Mixed methods	USA	4	NR	HD (HF)	+	−	+	−	−	−	−
Bernhard, 2018 [77]	Qualitative	Germany	25	64	DM	−^r^	−^r^	−^r^	−^r^	−^r^	−^r^	−
Coughlin, 2018 [78]	Quantitative	USA	455	NR	DM	−	−	−	−^r^	+	+	+
Dang, 2018 [79]	Quantitative	USA	120	64.8	HD (HF)	+	−	+	−	−	−	+
Lin, 2018 [80]	Quantitative	Taiwan	31	61	HD	+	−	−	−	−	−	+
Martinez, 2018 [81]	Mixed methods	USA	14	63	DM	+	+	−	−	−	+	+
Melholt, 2018 [82]	Quantitative	Denmark	49	60.6	HD	+	−	−	−	−	−	−
Poduval, 2018 [83]	Quantitative	UK	317	58.4	DM	+	−	−	−	+	−	+
Poppe, 2018 [84]	Mixed methods	Belgium	21	65.9	DM	+	+	+	−	−	−	−
Powell, 2018 [85]	Qualitative	USA	9	NR	DM, HD, CVD	−^r^	−^r^	−^r^	−^r^	−^r^	−^r^	−
Stamm-Balderjahn, 2018 [86]	Mixed methods	Germany	105	52	CVD	+	−	−	−	−	−	−
Tanaka, 2018 [87]	Mixed methods	Canada	13	63	HD, CVD	+	−	−	−	−	−	+
Torri, 2018 [88]	Quantitative	Italy	53	66	HD	+	−	+	+	−	−	+
Van Middelaar, 2018 [89]	Qualitative	Netherlands	20	70.8	DM, CVD	+	+	+	−	−	−	+
Wildenbos, 2018 [90]	Quantitative	Netherlands	1294	NR	HD	−^r^	−^r^	−^r^	−^r^	−^r^	−^r^	−
Casillas, 2019 [91]	Qualitative	USA	46	56.1	DM, CVD	+	−	−	−	+	+	+
Conway, 2019 [92]	Mixed methods	UK	1095	58	DM	+	−	+	+	+	+	−
Cunningham, 2019 [93]	Quantitative	UK	24,635	NR	DM	+	+	+	+	+	+	+
Joensson, 2019 [94]	Quantitative	Denmark	20	53	HD (HF)	−	−	+	+	−	−	+
Sun, 2019 [95]	Quantitative	USA	38,399	63.5	DM	−	−	−	−	+	+	+
Du Pont, 2020 [96]	Quantitative	Netherlands	193	69.9	DM	+	+	−	−	+	−	+
Ghisi, 2020 [97]	Mixed methods	Canada	84	59.8	DM	+	−	−	−	−	−	−
Poduval, 2020 [98]	Mixed methods	UK	791	57.6	DM	+	+	−	−	−	−	−
Powell, 2020 [99]	Quantitative	USA	500	66.4	HD, DM	−	−	+	−	−	+	+
Robinson, 2020 [100]	Quantitative	USA	446	66.4	DM	−	−	−	−	+	−	+
Signal, 2020 [101]	Mixed methods	New Zealand	215	NR	DM	+	−	−	−	−	−	+
Stewart, 2020 [102]	Qualitative	USA	40	65.9	DM	−	+	−	−	−	+	+
Clarke, 2021 [103]	Quantitative	USA	79	57-63^s^	HD	+	−	−	−	+	+	+
Conway, 2021 [104]	Quantitative	UK	55,605	NR	DM	+	+	+	+	+	+	+
Martinez, 2021 [105]	Mixed methods	USA	60	57.5	DM	+	−	+	+	+	+	+
Sabo, 2021 [106]	Quantitative	USA	337	61	DM	+	+	+	−	−	−	−
Fuji, 2022 [107]	Mixed methods	USA	117	59	DM	−	−	+	−	+	+	+
Holmes-Truscott, 2022 [108]	Quantitative	Australia	35	59-66^t^	DM	+	−	−	−	−	−	−
Litchman, 2022 [109]	Mixed methods	USA	31	54.5	DM	−	−	−	−	−	−	+

^a^Edu: educational/general information.

^b^R: reminder.

^c^M: monitoring.

^d^Int: interactive elements.

^e^PHI: personal health information.

^f^EHR/PHR: electronic/personal health record.

^g^C: communication.

^h^HD: heart disease.

^i^Available.

^j^Not available.

^k^DM: diabetes mellitus.

^l^Separate reporting for quantitative and qualitative analysis.

^m^ROK: Republic of Korea.

^n^CVD: cardiovascular disease.

^o^NR: not reported.

^p^HF: heart failure.

^q^CHD: coronary heart disease.

^r^Unclear due to insufficient description.

^s^Separate reporting for users (mean 57) and nonusers (mean 63).

^t^Separate reporting for intervention (mean 66) and control groups (mean 59).

### Usage and Usability of Patient Portals

In order to map aspects of usage behavior and usability as comprehensively as possible, we divided the results of the included studies into 7 categories. Table 3 provides an overview of the number of studies reporting each category of results.

**Table 3:** Reported outcomes in the included studies.

Outcome	Studies reporting quantitative results (N=73), n (%)	Studies reporting qualitative results (N=47), n (%)
Usage behavior	48 (66)	0 (0)
Associations with portal use	20 (27)	3 (6)
General usability	23 (32)	7 (15)
Satisfaction^a^	17 (23)	8 (17)
General benefits of use	0 (0)	20 (43)
Usefulness for self-management	14 (19)	17 (36)
Barriers to use	1^b^ (1)	31 (66)
Suggestions for improvement	5^b^ (7)	20 (43)

^a^We reported satisfaction separate from general usability, since this was often reported separate from usability in the included studies.

^b^Information on this was mostly collected with supplementary free-text responses in surveys.

### Usage Behavior

The usage behavior for portals was mostly reported using the login frequency, duration of a portal visit, and most used portal functions.

A total of 18 studies reported on the average number of logins within a month or a year [25,28,30,32,37,39,41,44,45, 48-50,60,65,66,80,89,99]. The data were provided either per user or as a total sum. The range of portal visits per user within 1 month was 0 to 7. Cunningham et al [93] reported an average of 8.8 logins per user per year. Four studies reported the average duration of a portal visit. The average time spent visiting the portal ranged from 7 to 53 minutes per portal visit [30,44,62,70].

A further 13 studies reported portal use in a highly heterogeneous manner. Three studies reported the total time spent using the portal. On average, this was 49 to 951 minutes over a time period of 2 to 6 months [80,84,96]. Kerr et al [27] reported a median of 4 logins in 9 months. Mayberry et al [31] reported that the use of the portal ranged from “sometimes” to “often.” Ronda et al [57] found that 53% of users logged in less than once a month. In addition, for half of the users, a portal visit took less than 15 minutes. Nelson et al [62] reported logins on 4.2 days in a fortnight. van Vugt et al [64] stated that 97% of participants logged into the portal at least once within 12 months, and 46% of participants logged in only once. Shaw et al [72] reported that 36% of participants used the portal within the last 30 days. Almost one-third of the population in the study by Sun et al [95] used the portal on a median of 31 days over a 2-year period. Holmes-Truscott et al [108] reported that the median time participants spent in the portal was 13.3 minutes (range, 3-28.6 minutes). In the study by Litchman et al [109], participants read portal content for an average of 2.4 hours (range, 0-13 hours). Martinez et al [105] reported that most participants visited the portal three or more times and half of them spent a total of ≥15 minutes on the portal.

In addition, 10 studies reported a decrease in activity or number of logins over time [32,37,49,50,63,66,67,98,101,106]. However, 2 studies reported a significant increase in the use of functions such as ordering medications, viewing laboratory results, and a health diary [63,79]. A total of 16 studies reported on the most used features or most beneficial elements of their portal (Multimedia Appendix 3) [28-30,32,33,35,36,62, 65,83,86,93,95,99,101,105]. These mainly concerned laboratory and test results, monitoring functions, and health information. Contrary to the most used features, 2 studies indicated the least used features or the least beneficial elements of the portal. These included participation in online support groups, sharing medical information with family members, and error aspects of the portal.

Furthermore, Nijland et al [32] reported more opportunities for self-care, more continuous feedback, and improved access to care as reasons for use, while a lack of the internet was reported as the main reason for not using the portal. Sievrink et al [50] reported that 34% of portal users clicked on their health scores first, 29% clicked on education, and 13% clicked on inbox. Jones et al [55] reported that the 2 largest of 8 groups logged into the portal infrequently overall, but they showed large differences in engagement with their respective health records. Wake et al [65] noted that despite positive feedback, only 5.7% of patients with DM in Scotland registered for the portal. Connelly et al [66] reported that only 2 of the portal features were used (goal setting function and log book).

#### Associations With Portal Use

In the 20 quantitative studies on variables associated with portal use, age was the most frequently cited variable (14/20, 70%), followed by gender (12/20, 60%), education (6/20, 30%), eHealth/health literacy (6/20, 30%), and specific reasons for interest or noninterest (3/20, 15%). Regarding age, younger age was frequently associated with a greater interest in using portals, and portal users were on average younger than nonusers [26,34,45,46,65,68,83,93,95,104]. However, Powell et al [99] reported an increase in the number of logins with increasing age. In addition, another study found that patients aged under 65 years valued secure logins and were reluctant to communicate with their providers through a portal [90].

With regard to the role of gender in portal use, most studies did not show significant differences. However, in 1 study, women more likely registered for the portal than men [78]. Moreover, 2 other studies reported that portal users were more likely to be male compared with nonusers [95,104]. Furthermore, in 3 studies, those interested in portals were more often male [46,96,104].

Six studies reported about the role of educational level regarding portal use or interest in a portal [26,46,47,63,68,95]. Five of these studies reported an association between a higher level of education and interest in or actual use of a portal [26,46,47,68,95]. Two further studies reported the presence of a social gradient with regard to portal use, with less deprived persons using the portal more often [92,93].

Six studies reported on the role of health or eHealth literacy. Sarkar et al [29] reported lower portal use among study participants with limited health literacy, as well as an association between limited health literacy and the likelihood of never signing up for a portal. Roelofsen et al [46] found that 71% of patients were interested in eHealth, of whom 42% registered for the portal and 27% of those registered used the portal. Melholt et al [82] reported no association between eHealth literacy and gender or age. However, an association was found between eHealth literacy and participants’ computer use or internet use [82]. Casillas et al [91] reported no significant differences in eHealth literacy scores between study participants of different languages. Clarke et al [103] reported a significantly higher computer self-efficacy in PHR users than in nonusers, but no difference regarding health literacy. Toscos et al [63] reported no significant difference between users and nonusers regarding internet use and ability to understand internet information.

Three studies reported on the reasons for portal use, reasons for interest in the portal, or reasons for disinterest in the portal. The main reasons for requesting a login to the portal were the ability to review information from the consultation and the ability to access laboratory results and treatment goals [47,57]. One study reported the main reason for not requesting login access as unawareness of the portal [47]. The reasons provided for not using the portal were preference for personal contact and computer problems [68].

The qualitative studies indicated that the interest of the portal provider in the portal, as perceived by the patient, and the hope that the portal would make the patient more aware of coping with the disease showed associations [37]. However, ignorance of the portal, not having access to a computer, and having a family member as an online delegate were cited as reasons for nonuse [40]. Furthermore, Powell et al [85] reported that participants had learned about the portal through nurses, doctors, or office staff.

### Usability

A total of 22 studies, including 13 involving a mixed-methods approach, reported quantitative results on the usability of portals [105]. User self-reports were used to assess usability in 18 studies, and of these, 9 used proprietary questionnaires [25,33,36,44,47,69,92,94,101] and the other 9 used validated questionnaires [43,59,62,71,74-76,100,105]. Studies used the System Usability Scale (SUS), Computer System Usability Questionnaire (CSUQ), and diabetes self-management questionnaire, as well as self-defined measurement techniques such as participation rates. Individual studies used other measures, such as participation rate per session or completion of >80% of the program [48,88], or did not report the measure [41,70].

Along with heterogeneous measurement techniques, the results of usability tests were reported very differently. Two-thirds of the measurements showed positive perceptions regarding the user friendliness, usability, and comprehensibility of online portals. For the remaining measurements, the scores were lower, with no study having a score lower than 50% of the respective scale. The average age of the participants in the studies reporting good usability ranged from 52 to 72 years, indicating no clear difference from studies reporting neutral or negative results on usability (52 to 70 years). However, 2 studies reporting insufficient usability did not mention the average age of their participants.

The 7 studies reporting qualitative results on usability largely confirmed the findings from the quantitative studies. The portals were overall perceived as useful, instructive, and easy to understand (Multimedia Appendix 4) [39,52,61,71,84,97,101].

### Satisfaction

Satisfaction was reported quantitatively in 18 studies [35,36,48,49,52,56,65,67,79-82,86,87,97,101,105,108]. Analogous to the measurement of usability, the most commonly used measurement methods were self-generated questionnaires. In 8 of the 18 studies, participant satisfaction was measured by elicited satisfaction or by participants’ positive comments [35,36,56,79-81,87,97]. Overall, satisfaction was consistently rated high (≥75%) or was reported across the board as satisfaction with the portal. Studies that created a portal prototype and later adapted it showed an increase in satisfaction or positive feedback from participants.

Four studies measured satisfaction via a possible recommendation of the portal. Recommendation rates ranged from 64% to 100%. A study showed that about 90% of the participants found the portal helpful, for example, to better manage diabetes or to improve knowledge and motivation [65]. Another study reported consistently positive comments and feedback on the portal’s acceptance, similar to another study in which the portal left a positive impression on participants [49,82].

The 8 studies reporting qualitative results on satisfaction largely confirmed the findings from the quantitative studies. Overall, satisfaction of the participants was high and the opinions were positive (Multimedia Appendix 4) [27,33,35,56,58,76,108,109].

### General Benefits of Portal Use

A total of 20 studies reported qualitatively on the benefits of portal use (Multimedia Appendix 5). Ten studies provided information on particularly popular features [33,37,44,51,54,71,73,85,92,105]. Particularly popular features included bidirectional communication, viewing laboratory results, and generally accessing comprehensive medical data and further information. Seven studies reported on satisfactory aspects (positively perceived portal effects) [42,53,58,60,91,98,102]. Improved communication and convenience of online access to medical resources were mainly mentioned. Four studies mentioned further benefits [31,73,89]. These included the possibility for family members to provide support in using the portal (social support), the benefits of secure messaging (asynchrony and recording of communications), integration into the daily routine, regular automated reminders, and monitoring.

### Usefulness for Self-Management

A total of 27 studies reported, at least in part, the extent to which portals were helpful for patient self-management. Of these studies, 13 reported quantitative results [25,44,48,49,52,64, 68,79,92,97,98,101,105] and 17 reported qualitative results [36,38,40,43,44,51,54,56,60,69,84,87,89,92, 101,102,107] (4 mixed-methods studies [36,44,92,101]).

Among the studies with quantitative results, 7 indicated a benefit, 3 indicated no benefit, and 3 indicated an unclear benefit of portal use for self-management. Individual studies used established self-management scales, namely the Self-care in Heart Failure Index (SCHFI) and the diabetes self-management self-efficacy scale (DSMSES), to examine the influence of portal use [25,52,98]. In this regard, Clark et al [52] and Poduval et al [98] found significant improvement in self-management, but Brennan et al [25] did not.

The studies that reported qualitatively on this aspect showed findings similar to those of the quantitative studies. Among the studies, 12 reported a benefit and 1 did not report a benefit. Moreover, in 2 studies, the benefit was unclear. Aspects regarding the benefits of portal use for self-management were general improvement in self-management [36,40,44,56,87,89,92], raising awareness of health [38,54,84,102], general enhancement of motivation [43,51], and assistance with behavior change [54,60,69,101]. In 1 study, the patients apparently saw no benefit from the portal for their self-management, which was partly due to computer problems and the existing use of other tools [107].

Across all 27 studies, providing general and educational information and personal monitoring were cited as common portal features helpful for self-management (8 studies each), followed by communication features such as a forum (6 studies) and access to individual personal health information (3 studies).

### Barriers of Portal Use

Two studies reported barriers of portal use using quantitative methodology. The most common barriers (mentioned by >20% of study participants) were unawareness of the portal, reading difficulties, limited computer literacy, needing help reading health-related materials, and not having their own computer [29,65].

Barriers were examined considerably more often in qualitative studies (31 studies). Reported barriers can be divided into general, specific, and unspecific detailed barriers. Four studies reported both general and specific barriers.

General barriers were reported most frequently (13 studies) and can be categorized into 4 topic areas: content, efficiency or relevance, technology, and design or usability [32,33,37,44,51,56,62,71,75,81,86,89,91]. Technology barriers and usability/design barriers were the most common barriers (each mentioned 7 times). For example, in the area of general technical problems, internet and computer problems were mentioned [54].

A total of 13 studies reported on specific barriers that relate to certain situations when using the portal (7 studies) [32,33,36,37,89,92,109], or problems or negative aspects with certain portal features (6 studies) [27,42,43,54,72,73] (Multimedia Appendix 6). Regarding certain situations when using the portal, difficulties with important processes (such as the initial login) or the finding and understanding of relevant information as well as poor provider engagement stood out. In terms of specific barriers affecting specific features, a major focus on communication stood out. Barriers were found in preconceived opinions, pronounced concerns, or negative experiences with the communication method.

Eleven studies described unspecific barriers that were neither user nor technology specific [38,53,58,60,74,75,85, 87,98,101,102,109]. Four barriers were more frequently reported. On the one hand, there was patient preference for face-to-face communication. On the other hand, a lack of technical knowledge or computer skills had a negative impact on the use of a portal. In addition, no internet or a poor internet connection represented a barrier. In part, the integration of a portal into everyday life was also perceived as difficult [109].

Measured by their frequency of mention, most barriers were related to design or usability aspects as well as technology. Barriers concerning the content of portals, the personal requirements of users, and efficiency or relevance occurred less frequently.

### Suggestions for Improvement

Three studies reported on suggestions for improvement using quantitative methodology. Suggestions were highly variable and concerned both general and specific portal aspects. They included expanded options for managing one’s own health online, including tracking health status [33], and access to medical records in general [33,65]. In addition, online clinic appointment setup, a reminder function for appointments [57], and online forums [65] were mentioned.

Suggestions for improvement were mainly collected through qualitative studies. Five studies reported on specific suggestions [32,35,40,76,86], 8 studies reported on more general suggestions [44,51,53,59,60,85,91,92], and 7 studies reported on both types of suggestions [38,69,77,101,105,108,109]. Suggestions were related to reminder features, interaction, education, usability, and user support. Suggestions in the areas of interactivity, education, and usability were mentioned most frequently. Suggestions regarding reminder features and user support were less frequent. More details can be found in Multimedia Appendix 7 and 8.

## Discussion

### Principal Findings

The importance of patient portals has increased significantly in recent years with the rise of self-management initiatives for chronic diseases. To investigate whether existing portals actually incorporate the needs of patients or are primarily technology-driven, we performed this scoping review using patients with DM or heart disease as an exemplary target group.

We identified 85 articles, which described aspects of usage behavior and usability of patient portals. Among the portals described, the most frequently represented characteristic was the provision of general or educational information. The most frequently reported outcome was usage behavior (including frequency and associations of use), followed by qualitatively recorded barriers of usage and quantitative aspects of usability. The population in the identified studies was predominantly middle-aged, and patients over 65 years were rather underrepresented. However, the prevalence of diabetes remains the highest in this group [110]. This limits the validity for age groups above 65 years. The needs and attitudes of older patients toward patient portals may differ from those of younger patients. As pointed out by some included studies, there were often both technological and personal barriers [59,89,102]. Moreover, a close person is not always available for proxy portal access. However, technological (eg, availability of computers) and relational aspects (need for technical and personal support), which were also highlighted by Wilson et al [111] in another review on older patients, could also be identified.

Most of the portals studied offered general or educational information, often combined with communication and monitoring functions. In addition, information and communication were often valued high by patients. Thus, existing portals seem to offer what patients need in terms of basic features. However, even if portals are user friendly, many barriers still seem to remain on the user side or in technology. Older patients in particular, who have less access to modern technology, are unlikely to feel that portals add any value to the management of their disease. In addition, few studies in our review initially evaluated the technical affinity of their participants. If this had been done, for example, necessary technical training for participants or their proxies could have been provided at an early stage and the nonuse or temporal decline in use of patient portals could have been decreased. However, the lack of knowledge about an online portal was also provided as a reason for nonuse, which confirms the findings of other studies on eHealth interventions [112,113].

The simplified categorization of portal features in our review might be associated with the fact that less frequently reported features are not considered separately in this framework. For example, 6 studies reported that their portals also included a payment function (bill payment and viewing payment history) [31,33,34,40,42,103]. However, only 3 studies reported evaluation results on this aspect [33,40,42]. Two studies reported that the feature was not used because of navigation problems [33] or lack of experience with using computers [40]. One study only reported that the function was rarely used [42]. Since all these studies are from the United States, it is not possible to mention whether this feature is also available in portals tethered to EHRs in other countries and how it is used and perceived by users. Other complementary features included providing questionnaires [94] and registering directly with lifestyle groups [89].

The mapping of the literature found was partly complicated by the inconsistent reporting of the studies, which is often a challenge in scoping reviews. Often, clear information about the population, specifically the present disease, and the portal assessed or the survey instruments used was missing. Therefore, we roughly categorized the results of the included studies. For example, we decided to present usability and satisfaction separately owing to the study presentation, which could also be seen differently, as user satisfaction can be understood as a subcategory of usability [114]. However, this is also a technical discussion, as the term usability is often used in general when testing IT applications, while in the medical context, it is more common to speak of patient satisfaction with the intervention. This is due to the different terminology of the subject-specific authorship. Similarly, the sometimes very heterogeneous and inaccurate presentation of portal types makes it difficult to compare them (eg, PHRs with EHRs). By definition, the data stored in a PHR can but does not have to originate from an EHR [115]. Unfortunately, it is often not clear from the articles, or not clear at all, where the data in PHRs come from (entered by health care providers, patients alone, or both) and what type of connection to an EHR or PHR the portals generally have. Thus, a differentiated evaluation of these portal types in our review does not appear to be reliable.

With regard to the acceptance of patient portals in the care of chronic illnesses, cultural differences are conceivable depending on countries and populations [116]. In addition to the personal characteristics of affected individuals, socialization, socioeconomic conditions, and the assessment of credibility vis-à-vis medical authorities also play major roles. The latter has just been demonstrated in the COVID-19 pandemic. Another factor in acceptance is access to medical services. The values are significantly higher in those states that are characterized by a large surface area and low population density, with access made more difficult by long distances, than in states with a small surface area and high supply density, with access being more likely to be close to home and close in time. Many of these aspects are not sufficiently addressed in the published studies and, according to the authors, often allow for direct comparability. Finally, the individual disease burden is also decisive in determining the degree of acceptance. This is also only peripherally taken into account.

The inclusion of quantitative, qualitative, and mixed-methods studies in a scoping review allows purely quantitative measures to be explained by qualitative findings. As expected, the qualitative studies provided deeper insights into the usability of portals compared to quantitative studies with predetermined narrowly defined response items. For example, some quantitative studies found a decline in portal use over time, and suggestions for improvement showed that there is a need for user support. It might be necessary to have a fixed contact person for problems, especially if the portal is used over a longer period of time. However, the paradigm can also lead to opposite results. For example, in the quantitative studies, usability was predominantly rated as good, while the qualitative studies revealed some weaknesses in this respect. When considering qualitative and quantitative approaches together, the possibilities and limitations of both approaches must always be taken into account.

### Limitations

This scoping review has some limitations in addition to the weaknesses in terminology and focus already pointed out. First, although there was some overlap, mobile apps were not part of this review. Mobile apps or mobile health (mHealth) apps are often used in research to describe the location and time of access, but not the research objectives such as usage behavior or user satisfaction. Therefore, studies may have been missed if the portals were developed for mobile and web use, but were only described as “mHealth” in the publications. Second, EHRs or PHRs, for which the terms “patient portal” or “online portal” are often used synonymously, were not systematically searched for. As the research objective was not focused on the usage and usability of EHRs as such, these terms were purposefully omitted. Third, studies that include patients with DM or CHD should be searched. In deviation from this, terms for the advanced forms of CHD (eg “chronic heart disease” and “heart failure”) were included in the search and selection, for example. This was due to inconsistent reporting, as we found in the initial searches.

### Future Research

Although literature about the usage patterns and usability of eHealth applications in general is growing rapidly, there are still some aspects that need to be addressed. Existing reviews suggest that higher disease burden is associated with higher portal use (for portals tethered to EHRs) [13]. We found insufficient data on this topic in our review and therefore could not make a statement on this. It is possible that disease burden affects portal usage differently for other portal types or different disease conditions. Therefore, future studies should consider the impact of disease burden (eg, diabetes without complications and heart disease) on portal usage behavior.

Other portal features that did not come up in this review but are of increasing importance are “personal device data integration” and “remote patient monitoring.” The search strategies in future reviews on these topics should broaden the “patient portal” to include the areas of telemedicine, monitoring systems, and integrative health applications.

In addition, since this was outside the scope of our review, future reviews should compare different portal types provided as mHealth apps. Moreover, a comparison of the usage patterns and usability of web-based portals with mHealth apps specifically in elderly patients with diabetes and cardiac issues would be desirable. Given the breadth of portal types and application areas expected, a scoping review approach is helpful.

### Conclusions

The current state of research emphasizes the importance of involving patients in the development and evaluation of patient portals. The consideration of quantitative, qualitative, and mixed-methods studies in a scoping review is helpful for developing a deeper understanding of usage behavior and usability. Future research should focus on the role of disease burden, and usage behavior and usability among older patients.

## Appendix

Multimedia Appendix 1 PRISMA-ScR (Preferred Reporting Items for Systematic Reviews and Meta-Analyses extension for Scoping Reviews) checklist.

Multimedia Appendix 2 Search strategies.

Multimedia Appendix 3 Most frequently used features or most beneficial elements of patient portals.

Multimedia Appendix 4 Qualitative results on usability and satisfaction.

Multimedia Appendix 5 Qualitative results on the benefits of portal use.

Multimedia Appendix 6 Qualitative results on the specific barriers of portal use.

Multimedia Appendix 7 Suggestions for improvement.

Multimedia Appendix 8 Frequency of the themes of suggestions for improvement.

## Abbreviations

CHD: coronary heart disease
DM: diabetes mellitus
EHR: electronic health record
mHealth: mobile health
PHR: personal health record
PRISMA: Preferred Reporting Items for Systematic Reviews and Meta-Analyses
