# Supplementary material for: Patient Portals as Facilitators of Engagement in Patients With Diabetes and Chronic Heart Disease: Scoping Review of Usage and Usability
Source: J Med Internet Res. 2023 Aug 25;25:e38447. doi: 10.2196/38447 (PMC10492174; doi:10.2196/38447)
Supplement: Multimedia Appendix 2 [file jmir_v25i1e38447_app2.docx]

**PubMed**

| **#** | **Query** |
| --- | --- |
| 1 | (patient portal [MeSH] OR patient portal* [TiAb] OR online portal* [TiAb] OR internet portal* [TiAb] OR web portal* [TiAb] OR information portal* [TiAb] OR consumer health information [MeSH] OR health information management [MeSH] OR electronic information [TiAb] OR online information [TiAb] OR web-based information [TiAb] OR online resource* [TiAb] OR electronic resource* [TiAb] OR internet resource* [TiAb] OR online tool* [TiAb] OR internet tool* [TiAb] OR internet-based tool* [TiAb] OR internet-based intervention [MeSH] OR online platform* [TiAb] OR internet platform* [TiAb] OR online community [TiAb] OR online forum [TiAb] OR web-based material [TiAb] OR web-based platform* [TiAb] OR internet-based information [TiAb] OR internet- based platform* [TiAb] OR internet-based portal* [TiAb] OR web-based tool* [TiAb] OR web-based portal* [TiAb] OR user interface* [TiAb] OR web interface* [TiAb] OR personal health record [TiAb] OR PHR [TiAb]) |
| 2 | (chronic disease [MeSH] OR chronic* [TiAb] OR diabetes [TiAb] OR diabetes mellitus, Type 2 [MeSH] OR CHD [TiAb] OR coronary heart disease [TiAb] OR heart disease [TiAb] OR coronary [TiAb] OR cardiovascular disease [TiAb] OR Cardiovascular Diseases [MeSH] OR heart failure [TiAb]) |
| 3 | (adopt*[TiAb] OR accept*[TiAb] OR Patient Acceptance of Health Care [MeSH] OR feasibility [TiAb] OR feasib*[TiAb] OR usability [TiAb] OR use [TiAb] OR user [TiAb] OR usage [TiAb] OR utilis*[TiAb] OR utiliz*[TiAb] OR usefulness [TiAb] OR practicab* [TiAb] OR facilitators [TiAb] OR barrier* [TiAb] OR registration[TiAb] OR login[TiAb] OR access* [TiAb] OR self management [TiAb] OR Self-Management [MeSH] OR satisf*[TiAb] OR Patient Satisfaction [MeSH] OR compliance [TiAb] OR Patient Compliance [MeSH] OR enrollment [TiAb]) |
| 4 | 1 AND 2 AND 3 |

Limits: 2010-2021

Search was updated 29/04/2021 and 30/11/2022.

**PsycINFO (via EbscoHost)**

| **#** | **Query** | **Limiters/Expanders** |
| --- | --- | --- |
| S1 | TI ( patient portal* OR online portal* OR internet portal* OR web portal* OR information portal* OR electronic information OR online information OR web-based information OR online resource* OR electronic resource* OR internet resource* OR online tool* OR internet tool* OR internet-based tool* OR online platform* OR internet platform* OR online community OR online forum OR web-based material OR web-based platform* OR internet-based information OR internet-based platform* OR internet-based portal* OR web-based tool* OR web-based portal* OR web interface* OR user interface* OR personal health record* OR PHR) OR AB ( patient portal* OR online portal* OR internet portal* OR web portal* OR information portal* OR electronic information OR online information OR web-based information OR online resource* OR electronic resource* OR internet resource* OR online tool* OR internet tool* OR internet-based tool* OR online platform* OR internet platform* OR online community OR online forum OR web-based material OR web-based platform* OR internet-based information OR internet-based platform* OR internet-based portal* OR web-based tool* OR web-based portal* OR web interface* OR user interface* OR personal health record* OR PHR) OR DE ( health information technology OR digital interventions OR Human Computer Interaction OR personal health record* OR PHR) | Expanders - Apply related words; Apply  equivalent subjects  Search modes - Boolean/Phrase |
| S2 | TI ( chronic* OR diabetes OR CHD OR coronary heart disease OR heart disease OR coronary OR cardiovascular disease OR heart failure ) OR AB ( chronic* OR diabetes OR CHD OR coronary heart disease OR heart disease OR coronary OR cardiovascular disease OR heart failure ) OR DE ( chronic illness OR diabetes OR cardiovascular disorders ) | Expanders - Apply related words; Apply  equivalent subjects  Search modes - Boolean/Phrase |
| S3 | TI ( adopt* OR accept* OR feasibility OR feasib* OR usability OR use OR user ORusage OR usefulness OR utilis* OR utiliz* OR practicab* OR facilitator* OR barrier* OR registration OR login OR access* OR self management OR satisf* OR compliance OR enrollment ) OR AB ( adopt* OR accept* OR feasibility OR feasib* OR usability OR use OR user OR usage OR usefulness OR utilis* OR utiliz* OR practicab* OR facilitator* OR barrier* OR registration OR login OR access* OR self management OR satisf* OR compliance OR enrollment ) OR DE ( Self- Management OR client satisfaction OR Treatment Compliance ) | Expanders - Apply related words; Apply equivalent subjects  Search modes - Boolean/Phrase |
| S4 | S1 AND S2 AND S3 | Expanders - Apply equivalent subjects; Search modes - Find all my search terms |
| S5 | S1 AND S2 AND S3 | Limiters - Publication Year: 2010-2020*; Expanders - Apply equivalent subjects |

Search was updated 29/04/2021 and 30/11/2022.

**CINAHL (via Ovid)**

| **#** | **Query** | **Limiters/Expanders** |
| --- | --- | --- |
| S1 | (MH "Patient Portals") | Expanders - Apply equivalent subjects  Search modes - Boolean/Phrase |
| S2 | (MH "personal health records") | Expanders - Apply equivalent subjects  Search modes - Boolean/Phrase |
| S3 | TX (personal health record* OR PHR) | Expanders - Apply equivalent subjects  Search modes - Boolean/Phrase |
| S4 | TX ((patient* OR online OR internet OR web OR information) N2 portal) | Expanders - Apply equivalent subjects  Search modes - Boolean/Phrase |
| S5 | TX ((patient* OR online OR internet OR web OR information) N2 portal*) | Expanders - Apply equivalent subjects  Search modes - Boolean/Phrase |
| S6 | (MH "Health Information Management") | Expanders - Apply equivalent subjects  Search modes - Boolean/Phrase |
| S7 | TX (electronic information OR online information OR web-based information OR online resource* OR electronic resource* OR internet resource* OR online tool* OR internet tool* OR internet-based tool*) | Expanders - Apply equivalent subjects  Search modes - Boolean/Phrase |
| S8 | (MH "Chronic Disease+") | Expanders - Apply equivalent subjects  Search modes - Boolean/Phrase |
| S9 | (MH "Diabetes Mellitus, Type 2") | Expanders - Apply equivalent subjects  Search modes - Boolean/Phrase |
| S10 | (MH "Cardiovascular Diseases+") | Expanders - Apply equivalent subjects  Search modes - Boolean/Phrase |
| S11 | TX (chronic* OR diabetes OR CHD OR coronary heart disease OR heart disease OR coronary OR cardiovascular disease OR heart failure) | Expanders - Apply equivalent subjects  Search modes - Boolean/Phrase |
| S12 | (MH "Patient Attitudes") | Expanders - Apply equivalent subjects  Search modes - Boolean/Phrase |
| S13 | (MH "Health Services Accessibility+") | Expanders - Apply equivalent subjects  Search modes - Boolean/Phrase |
| S14 | S10 AND S11 | Expanders - Apply equivalent subjects  Search modes - Boolean/Phrase |
| S15 | (MH "Self-Management") OR (MH "Self Care+") | Expanders - Apply equivalent subjects  Search modes - Boolean/Phrase |
| S16 | (MH "Patient Satisfaction+") | Expanders - Apply equivalent subjects  Search modes - Boolean/Phrase |
| S17 | (MH "Patient Compliance+") OR (MH "Attitude to Health+") | Expanders - Apply equivalent subjects  Search modes - Boolean/Phrase |
| S18 | (adopt* OR accept* OR OR feasibility OR feasib* OR usability OR use OR user OR usage OR utilis* OR utiliz* OR usefulness OR practicab* OR facilitators OR barrier* OR registration OR login OR access* OR self management OR satisf* OR compliance OR enrolment) | Expanders - Apply equivalent subjects  Search modes - Boolean/Phrase |
| S19 | S1 OR S2 OR S3 OR S4 OR S5 OR S6 OR S7 | Expanders - Apply equivalent subjects  Search modes - Boolean/Phrase |
| S20 | S8 OR S9 OR S10 OR S11 OR S12 OR S13 | Expanders - Apply equivalent subjects  Search modes - Boolean/Phrase |
| S21 | S14 OR S15 OR S16 OR S17 OR S18 | Expanders - Apply equivalent subjects  Search modes - Boolean/Phrase |
| S22 | S19 AND S20 AND S21 | Expanders - Apply equivalent subjects  Search modes - Boolean/Phrase |
| S23 | S19 AND S20 AND S21 | Limiters - Published Date: 20090101-20200731*  Expanders - Apply equivalent subjects  Search modes - Boolean/Phrase |

Search was updated 29/04/2021 and 30/11/2022.

Hand searches:

| Databases | - Google Scholar - IEEE Xplore |
| --- | --- |
| Trial registries | - Clinicaltrials.gov - DRKS (Germany) |
| Journals | - Journal of Medical Internet Research - Health and Technology - Digital Health - JAMIA (Journal of the American Medical Informatics Association - JMIR Diabetes - Diabetes Care - BMC Medical Informatics and Decision Making - Journal of Diabetes Science and Technology - Diabetes Technology and Therapeutics - Journal of Biomedical Informatics - Telemed Journal and E -Health - Journal of the American Medical Informatics Association - International Journal of Human-Computer Interaction - International Journal of Medical Informatics - Health Informatics |
| Reference lists | - All included articles - All identified reviews from the database searches |

Search was updated 29/04/2021 and 30/11/2022.
