# Supplementary material for: Patient Portals as Facilitators of Engagement in Patients With Diabetes and Chronic Heart Disease: Scoping Review of Usage and Usability
Source: J Med Internet Res. 2023 Aug 25;25:e38447. doi: 10.2196/38447 (PMC10492174; doi:10.2196/38447)
Supplement: Multimedia Appendix 3 [file jmir_v25i1e38447_app3.docx]

**Multimedia Appendix 3.** Most frequently used features or most beneficial elements of patient portals.

| Outcome | Feature/element |
| --- | --- |
| Most frequent used function or most beneficial element | General health information / information about own diseases [28,33,35,93,101]  View lab results [29] or test results [65,93]  Self-Monitoring [30,32]  Additional information [36]  Comprehensibility of the information (perceived as beneficial) [62]  Health record [83] or medical summaries [95]  Search function [86]  Secure messaging [99]  health data infographic and literacy level–appropriate educational links [105] |

Reported by 16 studies.
