# Supplementary material for: Patient Portals as Facilitators of Engagement in Patients With Diabetes and Chronic Heart Disease: Scoping Review of Usage and Usability
Source: J Med Internet Res. 2023 Aug 25;25:e38447. doi: 10.2196/38447 (PMC10492174; doi:10.2196/38447)
Supplement: Multimedia Appendix 4 [file jmir_v25i1e38447_app4.docx]

**Multimedia Appendix 4.** Qualitative results on usability and satisfaction.

| Outcome | Aspects of usability and satisfaction |
| --- | --- |
| Usability | 82% positive reactions to the portal, majority finds portal useful [61]  Portal very easy to understand [52]  90% find the portal informative and educational [71]  moderate satisfaction with user-friendliness and the usefulness of the portal's functions [39]  In general satisfaction with the usability of the portal, easy to navigate, questions short and understandable [84]  Portal perceived as informative, useful, interesting and helpful [97]  Participants found the portal useful overall [101] |
| Satisfaction | Perception of the portal among participants very different, generally positive [27]  All participants will consider using the portal or recommend it to others [33]  88% are willing to use the portal to manage their health care [58]  All participants expressed themselves overall very positively about the portal [56]  Participants expressed satisfaction with the portal [35]  Participants expressed positive and negative impressions, overall liked the portal [76]  One third of responses (n = 29) indicated general satisfaction with the intervention and/or no comments for improvement [108]  The majority of participants were satisfied with the OPSC [109] |

Usability was reported by 7 studies and satisfaction by 8 studies.
