# Supplementary material for: Patient Portals as Facilitators of Engagement in Patients With Diabetes and Chronic Heart Disease: Scoping Review of Usage and Usability
Source: J Med Internet Res. 2023 Aug 25;25:e38447. doi: 10.2196/38447 (PMC10492174; doi:10.2196/38447)
Supplement: Multimedia Appendix 5 [file jmir_v25i1e38447_app5.docx]

**Multimedia Appendix 5.** Qualitative results on the benefits of portal use.

| Cathegory | Benefits |
| --- | --- |
| Particularly popular function | Making appointments and viewing laboratory results [33]  Bidirectional communication with the provider [37]  Goal-oriented use [51]  Live interactive lessons [71]  View communication and results [73]  Viewing lab results, requesting medication refills Scheduling appointments  Secure communication. [85]  Access to complete medical record [92]  Informational resources and social interaction [44]  Monitoring (PHR facilitated tracking additional types of health information) [54]  Health data infographic  Patients like me (display of average values)  Educational links [105] |
| Satisfactory aspects | Improved patient satisfaction  Improved efficiency and quality of face-to-face visits  Access to clinical care outside of traditional face-to-face visits [42]  Improved care with follow-up  Reduced uncertainty in the care plan  Automatic 'health archives' [53]  Comfort  Health monitoring  Improvements in communication between patients and providers [58]  Access to medical records  Access to laboratories or tests  Better medication adherence as a result of easier Medication refills  Less time spent on phone calls and transportation  Fewer missed appointments [91]  Feeling better informed and more aware  Taking diabetes more seriously  Increased self-efficacy and support  Improved handling of worries and dejection  Changes in self-management [60]  Practical format  Emotional support [98]  Increased commitment to healthcare [102] |
| Further benefits | Family members facilitate use of the portal  Family members help and teach patients how to use the portal [31]  Advantages of secure messaging: Asynchronous communication and electronic recording of communication [73]  Relationship of trust with coach  regular automatic and personal reminders  integration into the daily routine  social support [89] |

Reported by 20 studies.
