# Supplementary material for: Patient Portals as Facilitators of Engagement in Patients With Diabetes and Chronic Heart Disease: Scoping Review of Usage and Usability
Source: J Med Internet Res. 2023 Aug 25;25:e38447. doi: 10.2196/38447 (PMC10492174; doi:10.2196/38447)
Supplement: Multimedia Appendix 6 [file jmir_v25i1e38447_app6.docx]

**Multimedia Appendix 6.** Qualitative results on the specific barriers of portal use.

| Cathegory | Barriers |
| --- | --- |
| Related to specific situations while using the portal | Lack of a reminder function [32]  Lack of explanation of medical terminology [33]  Lack of commitment of the provider to the portal [37]  Lack of coach continuity  Setting a goal is perceived as burdensome  Problems with motivation to change lifestyle with increasing age [89]  Difficulties with the first login [92] and to remember where and how to log in [109]  Difficulty finding information about drugs and certain aspects of nutrition [36] |
| Related to specific features | Problems with self-monitoring [43]  Low view rate of the explainer video [72]  Portal is perceived as a more difficult, less personal and less effective means of communication [27]  Preconceived beliefs or rules about secure messaging  Previous negative experiences with secure messaging [42]  Concerns about secure messaging regarding:  Imposed work for the physician  Provider compensation  Confusion about timing of utilization [73]  Concerns about the privacy and security of information maintained in the PHR [54] |

Reported by 13 studies.
