# Supplementary material for: Patient Portals as Facilitators of Engagement in Patients With Diabetes and Chronic Heart Disease: Scoping Review of Usage and Usability
Source: J Med Internet Res. 2023 Aug 25;25:e38447. doi: 10.2196/38447 (PMC10492174; doi:10.2196/38447)
Supplement: Multimedia Appendix 7 [file jmir_v25i1e38447_app7.docx]

**Multimedia Appendix 7.** Suggestions for improvement.

| Cathegory | Suggestions |
| --- | --- |
| Specific suggestions | Notifications about new announcements  Interactive calendar  Scheduling via the portal [32]  Online tutorial [38]  Reminder function for refilling medications  Information on side effects and drug interactions [40]  Online interaction with other patients [69]  Security Alerts  Reminders  Notifications  Medication history tracking  Help section  Electronic messaging and information exchange [77]  Information on support services (e.g. self-help groups) in the area  Comprehensive information about underlying heart disease [86]  Exercises specifically tailored to target groups (e.g. older people)  Explanation of different foods  Explanation of the food pyramid  Explanation of different classes of diabetes medications [35]  Page for recording blood pressure values  Option link to set a time for automatic logout [76]  Face-to-face coaching instead of online coaching  Keeping the same coach throughout the program  Using coaches who can serve as role models  Connecting participants with each other  Involving the family  Counseling role [101]  provide synchronous sessions to improve group dynamic [109]  more specific clinical information (e.g. when insulin will be prescribed, dosage requirements) [108]  ability to upload blood sugar readings  integrating into the patient portal mobile app  refining the ruler infographic  depicting improvement or worsening of values (e.g. A1C) [105] |
| More general suggestions | Improved technical support [38]  social interactions  Information material  Usability  Diabetes knowledge  Community resources [44]  Better access to timely and personalized care  sharing experiences with other patients  patient-centered perspective in content presentation [51]  Incorporation of patient preferences  Status display system for individual requests [53]  Training and practice (familiarize with portal)  Support for at home [59]  more guidance [69]  clear and logical user interface  user-centered provision of information [77]  more user-friendly interface [85]  Improvement of portal registration  Ways to use portal easily [91]  additional educational resources [92]  Better support and training  improvements in functionality  more contact with health coach  increase of program flexibility [101]  Support with dietary changes and weight loss  help with mood swings  learn from other people with diabetes  more information about diabetes [60]  Improvement of print and search functionality  Additional hard copy resource [108]  Simpler platform  simpler explanations [109]  creating a walkthrough to orient new users  more diet information  updating information as new therapies emerge [105] |

Reported by 20 studies
