# Supplementary material for: Patient Portals as Facilitators of Engagement in Patients With Diabetes and Chronic Heart Disease: Scoping Review of Usage and Usability
Source: J Med Internet Res. 2023 Aug 25;25:e38447. doi: 10.2196/38447 (PMC10492174; doi:10.2196/38447)
Supplement: Multimedia Appendix 8 [file jmir_v25i1e38447_app8.docx]

**Multimedia Appendix 8.** Frequency of the themes of suggestions for improvement.

Frequency of mention across all 20 studies, which reported on suggestions of improvement
